# Classification of expert-level therapeutic decisions for degenerative cervical myelopathy using ensemble machine learning algorithms

**DOI:** 10.3389/fsurg.2022.1010420

**Published:** 2022-09-06

**Authors:** Dougho Park, Jae Man Cho, Joong Won Yang, Donghoon Yang, Mansu Kim, Gayeoul Oh, Heum Dai Kwon

**Affiliations:** ^1^ Department of Rehabilitation Medicine, Pohang Stroke and Spine Hospital, Pohang, South Korea; ^2^ Department of Neurosurgery, Pohang Stroke and Spine Hospital, Pohang, South Korea; ^3^ Department of Radiology, Pohang Stroke and Spine Hospital, Pohang, South Korea

**Keywords:** cervical spondylosis, myelopathy, machine learning, decision making, therapeutic options

## Abstract

**Background:**

Therapeutic decisions for degenerative cervical myelopathy (DCM) are complex and should consider various factors. We aimed to develop machine learning (ML) models for classifying expert-level therapeutic decisions in patients with DCM.

**Methods:**

This retrospective cross-sectional study included patients diagnosed with DCM, and the diagnosis of DCM was confirmed clinically and radiologically. The target outcomes were defined as conservative treatment, anterior surgical approaches (ASA), and posterior surgical approaches (PSA). We performed the following classifications using ML algorithms: multiclass, one-versus-rest, and one-versus-one. Two ensemble ML algorithms were used: random forest (RF) and extreme gradient boosting (XGB). The area under the receiver operating characteristic curve (AUC-ROC) was the primary metric. We also identified the variable importance for each classification.

**Results:**

In total, 304 patients were included (109 conservative, 66 ASA, 125 PSA, and 4 combined surgeries). For multiclass classification, the AUC-ROC of RF and XGB models were 0.91 and 0.92, respectively. In addition, ML models showed AUC-ROC values of >0.9 for all types of binary classifications. Variable importance analysis revealed that the modified Japanese Orthopaedic Association score and central motor conduction time were the two most important variables for distinguishing between conservative and surgical treatments. When classifying ASA and PSA, the number of involved levels, age, and body mass index were important contributing factors.

**Conclusion:**

ML-based classification of DCM therapeutic options is valid and feasible. This study can be a basis for establishing generalizable ML-based surgical decision models for DCM. Further studies are needed with a large multicenter database.

## Introduction

Degenerative cervical myelopathy (DCM) is a disease that causes progressive and nontraumatic cervical spinal cord compression due to degenerative changes in the cervical spine (1). Patients with DCM present a broad spectrum of symptoms, ranging from subtle sensory neuropathic symptoms to motor weakness with functional disability, depending on disease progression and severity (2). Patients with DCM require early diagnosis and management; specifically, it is essential to determine an appropriate therapeutic option according to the disease severity while minimizing damage to the spinal cord (3, 4).

There have been studies on determining the appropriate treatment options for DCM (5). A randomized controlled study of patients with mild-to-moderate DCM (modified Japanese Orthopaedic Association [mJOA] score >12) conducted by Kadanke et al. (6) showed that surgical treatment was not superior to conservative treatment. In contrast, studies have also suggested that surgical treatment demonstrated better functional recovery and patient satisfaction in patients with more severe DCM (7). In the guidelines presented in 2017, Fehlings et al. (8) argued that early diagnosis and surgical treatment are necessary for moderate-to-severe DCM. On the other hand, they reported that there was a knowledge gap in the selection of therapeutic options for mild DCM. Meanwhile, studies on the optimal surgical approach method in patients with DCM who decided to undergo surgery have also been reported (9). Several studies have compared outcomes after anterior and posterior surgical approaches (ASA and PSA, respectively) for DCM; however, no clear evidence of superiority has been established to date (5). A systematic review also concluded that there are no apparent differences between surgical methods in terms of neurologic recovery (10). Consequently, there has been a debate regarding the therapeutic decision for DCM (11). In addition, the surgical indications can be slightly different depending on the surgeon's practice style, preference, and health insurance system (12, 13).

Machine learning (ML) algorithms are actively applied to research for medical decisions because of their excellent classification and prediction (14). Specifically, ML algorithms can handle data with huge samples and use clinical information to make the medical decision-making process more efficient (15). In line with this, Park et al. (16) presented a disease severity classification model to minimize unnecessary electrodiagnostic testing in patients with carpal tunnel syndrome. Yoo et al. (17) reported an ML decision model for the selection of an optimal laser refractive surgery method. In addition, studies have applied ML to DCM. Merali et al. (18) predicted the 6-, 12-, and 24-month outcomes in 757 patients with DCM and showed an area under the receiver operating characteristic curve (AUC-ROC) of approximately 0.70. Hopkins et al. (19) presented models predicting DCM diagnosis and mJOA scores using a deep neural network in 14 patients with DCM and 14 healthy controls. In these studies, ensemble ML algorithms presented valid and accurate results, proving their clinical usefulness. As a result, ML-related research in the clinical field has been growing rapidly. Nevertheless, an ML model that classifies the therapeutic decisions in DCM has not yet been reported to the best of our knowledge.

In this context, we developed an ML-based model for classifying expert-level therapeutic decisions using ensemble ML algorithms in patients with DCM to verify their performance. In addition, we investigated the contributing factors involved in the therapeutic decisions in DCM through feature importance results derived from optimal ML classification models.

## Materials and methods

### Study design

This single-center, retrospective study enrolled patients diagnosed with DCM between January 2017 and December 2021. The dataset included patients of experienced neurosurgeons in our hospital who also co-authored this study. This study was reviewed and approved by the institutional review board of Pohang Stroke and Spine Hospital (PSSH-0475-202202-HR-007-01). It was performed in compliance with the Declaration of Helsinki and the International Conference on Harmonization-Good Clinical Practice Guidelines. Informed consent was not required owing to the retrospective nature of the study design.

The diagnosis of DCM was confirmed when it satisfied the following criteria: clinical manifestation, functional level, and cervical cord compression grade on magnetic resonance imaging (MRI). We utilized patient information—demographic, clinical, radiological, and electrodiagnostic characteristics—as variables for ML classification based on electrical health records (EHR). Therefore, variable definitions were primarily based on formats recorded in the EHR. Detailed information on each variable is provided in Supplementary Table S1.

**Figure 1:**
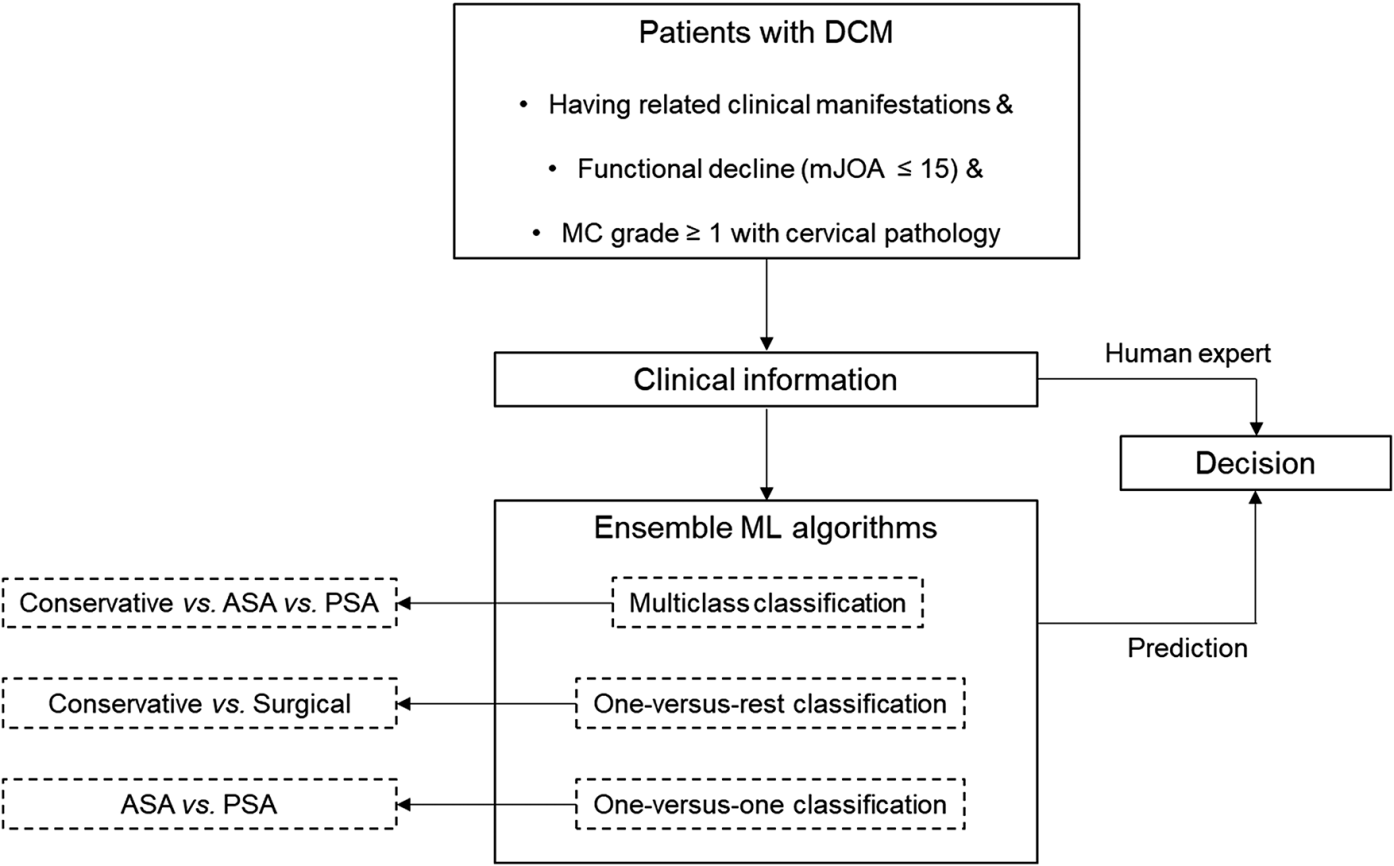
Schematic architecture of the study design. ASA, anterior surgical approaches; DCM, degenerative cervical myelopathy; MC, Muhle’s classification; mJOA; modified Japanese Orthopaedic Association score; PSA, posterior surgical approaches.

We performed an ML algorithm-based ensemble classification using the extracted variables. The ground truth was set based on an experienced neurosurgeon's decision. We proceeded with three types of classification—multiclass, one-versus-rest, and one-versus-one—according to the target outcomes (Figure 1).

### Patients and clinical assessments

DCM-related clinical manifestations were defined as follows: presence of upper motor neuron signs, clumsy hands, motor weakness, atrophic muscle change, progressive gait disturbance, sensory loss with altered proprioception, paresthesia, and bowel or bladder symptoms. Patients with at least one or more symptoms and signs, and a functional decline of ≤15 on the mJOA score were included in the confirmed cases of DCM. An experienced neurosurgeon or physiatrist enquired and evaluated the patient's symptoms and recorded the score. The exclusion criteria for this study were as follows: (1) brain lesion or brain surgery history, (2) history of cervical spine surgery, (3) previous thoracic myelopathy or cauda equina syndrome, (4) cervical cord compression lesion lower than the C6/7 level, (5) severe carpal tunnel syndrome, and (6) missing values.

During the first visit, in the period in which DCM was diagnosed, we measured the patient's numerical rating scale (NRS) of the neck and arm pain and recorded the subjective symptom duration in months. We identified comorbidities *via* patient or guardian interviews, medical records, and medication history.

The target classes were conservative treatment, ASA, and PSA. ASA included anterior cervical discectomy and fusion, anterior cervical corpectomy and fusion, and cervical arthroplasty. PSA included laminoplasty and laminectomy, with or without fusion. Considering poor compliance or transferred patients, we set the ground truth based on the decision of human experts, not on whether the actual surgery was performed.

### Radiologic features

Cervical MRI scans were performed using a 1.5 T Philips Achieva (Philips Medical Systems, Eindhoven, Netherlands). Imaging findings were analyzed at the time of DCM diagnosis and re-reviewed by a radiologist for this study as well. In case of discrepancy during the retrospective review, it was finally decided by the consensus of the radiologists and surgeon. The Muhle's classification (MC) grade, which was interpreted based on the most stenotic level in the midsagittal view of the MRI, was used as an index for cervical spinal cord compression (20). In addition, we counted the number of levels with an MC grade of I or higher. Lesion types were classified as ossification of the posterior longitudinal ligament (OPLL), disc herniation, spondylolisthesis, or others (including combined pathologies). We also identified the presence of high signal intensity (HSI) on T2 MRI.

We drew the k-line in the midline of the sagittal view of the cervical radiograph, which was recorded in the standing state to confirm cervical spine alignment (21). The k-line evaluation using a radiograph was retrospectively reviewed by an experienced radiologist and neurosurgeon. In case of discrepancy, it was finally decided by the consensus of the two interpreters.

### Electrodiagnostic evaluation

All electrodiagnostic tests were performed using a Sierra Wave (Cadwell Laboratories Inc., Kennewick, WA, USA). We conducted transmagnetic stimulation using MagPro Compact and a circular coil with an outer diameter of 12 cm (MagVenture Inc., Alpharetta, GA, USA). Stimulation for motor-evoked potential (MEP) was performed at the cortical and cervical levels and recorded from the abductor pollicis brevis muscle. Cortex stimulation was applied to the Cz region according to the international 10–20 system, and cervical stimulation was performed at the spinous process at the C7 level to obtain peripheral motor conduction time. The central motor conduction time (CMCT) was then calculated as follows (22):


CMCT(ms)=corticalMEPonsetlatency−cervicalMEPonsetlatency.

We used the CMCT value as a variable for the symptomatic side and a slower CMCT value for bilateral symptoms. CMCT was divided into four categories: normal, <11.5 ms; mildly delayed, 11.5 ≤ CMCT <15; definitely delayed, ≥15; and not evoked MEP.

We evaluated the upper extremity nerve conduction study and electromyography in all patients for differential diagnosis and identified radiculopathy, which was confirmed by electromyography. We diagnosed concomitant radiculopathies with denervation potentials in two or more muscles innervated by different peripheral nerves in the specific myotome (23). All electrodiagnostic tests were performed and interpreted by experienced physiatrists.

### Statistical analysis

We used R software version 4.1.3 (R Core Team, R Foundation for Statistical Computing, Vienna, Austria) and GraphPad Prism 9.3.1 (GraphPad Software, San Diego, CA, USA) for statistical analyses. Continuous variables were analyzed using Shapiro–Wilk normality test and expressed as mean ± standard deviation or median (interquartile range). Categorical variables are expressed as frequency (proportion). For comparative analysis between the conservative, ASA, and PSA groups, one-way analysis of variance with Bonferroni's multiple comparison test or Kruskal-Wallis test with Dunn’s multiple comparisons test was applied to continuous variables, and chi-squared trend test was used for categorical variables. Statistical significance was set at *p* < 0.05.

### Machine learning processing

**Figure 2:**
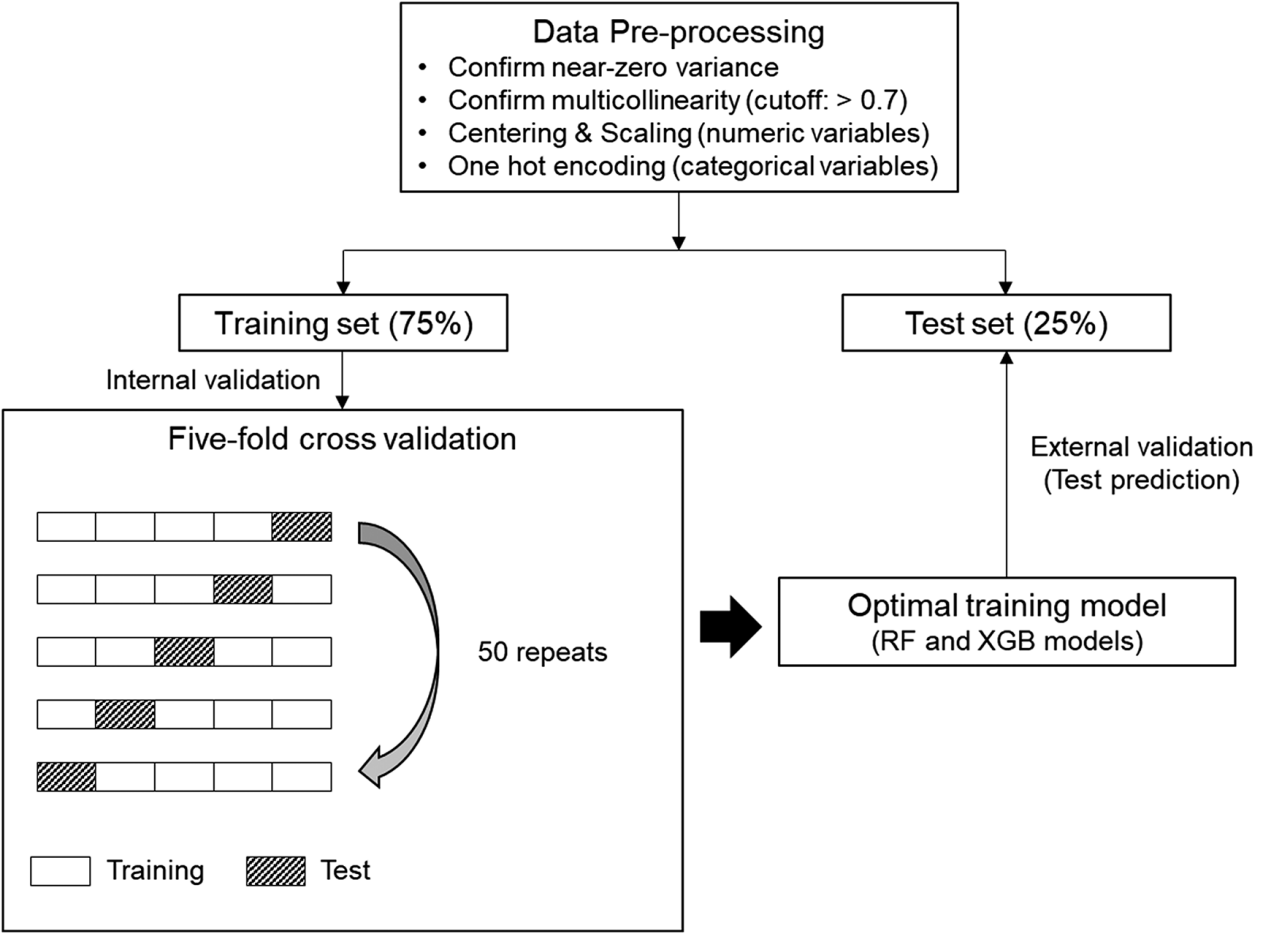
Machine learning (ML) process for this study. Five-fold cross-validation was repeated 50 times on the preprocessed training set to generate an optimal training model. Afterwards, test prediction was performed on the test set with the training model of each ML algorithm. RF, random forest; XGB, extreme gradient boosting.

ML modeling was performed based on the “caret” package of the R software (24). The ML processing in this study is shown in Figure 2. The R code in this study is available in the Online supplementary material. We confirmed variables with near-zero variance and multicollinearity (correlation coefficient >0.7) for variable selection. Subsequently, centering and scaling were performed for numeric variables, and one-hot encoding was performed for categorical variables.

The whole dataset was randomly split into 75%-training and 25%-test sets. In the binary classifications, the target class-balanced training set was additionally formed by applying the synthetic minority oversampling technique, after which we drove the ML algorithms. Random forest (RF) and extreme gradient boosting (XGB), which are representative ensemble ML algorithms, were used (25, 26). Five-fold cross-validation was repeated 50 times to generate the optimal training model. Random and grid search methods were applied for hyperparameter tuning. The tuned hyperparameter values applied to each classification are presented in Supplementary Table S2. External validation was performed by applying the training model generated for each algorithm to the test set. AUC-ROC and overall accuracy were used as the main metrics for multiclass classification. In the multiclass classification, AUC-ROC was defined as the micro-average value calculated by converting the multiclass into the sum of binary classifications using the “multiROC” package of R software (27). In the binary classifications, AUC-ROC, F1 measure, and area under the precision-recall curve (AUC-PR) were evaluated using the “MLeval” package of the R software (28).

## Results

### Baseline characteristics

A total of 304 patients were included (109 conservative and 195 surgical treatments). ASA was chosen in 66 patients, PSA in 125, and a combined approach was selected as the surgical treatment in four patients.

The PSA group was significantly older than the other two groups (*p* < 0.001), and the proportion of men was relatively higher (*p* = 0.023). In addition, the proportion of patients covered by medical-aid and living in rural areas in the PSA group was significantly higher (*p* = 0.045 and *p* < 0.001, respectively). Meanwhile, the ASA group showed a significantly lower body mass index (BMI) value of 23.5 (21.3–25.3) kg/m^2^ compared to 24.6 (23.0–26.6) kg/m^2^ in the PSA group (*p* = 0.036) (Table 1 and Supplementary Table S3).

**Table 1:** Baseline characteristics.

	Conservative (*n* = 109)	ASA (*n* = 66)	PSA (*n* = 125)	*p*-value
Age, years	55.9 ± 11.4	57.9 ± 13.1	64.0 ± 10.9	<0.001
Male, *n* (%)	61 (56.0)	40 (60.6)	91 (72.8)	0.023
BMI, kg/m^2^	24.0 (22.2–26.7)	23.5 (21.3–25.3)	24.6 (23.0–26.6)	0.036
Medical-aid, *n* (%)	3 (2.8)	2 (3.0)	12 (9.6)	0.045
Urban residence, *n* (%)	75 (68.8)	39 (59.1)	53 (42.4)	<0.001
Comorbidities, *n* (%)				
Hypertension	34 (31.2)	25 (37.9)	54 (43.2)	0.167
Diabetes	15 (13.8)	27 (21.6)	13 (19.7)	0.287
Dyslipidemia	14 (12.8)	9 (13.6)	20 (16.0)	0.777
Heart problems^a^	5 (4.6)	0 (0.0)	10 (8.0)	0.053
Degenerative lumbar disease	28 (25.7)	23 (34.8)	56 (44.8)	0.010

ASA, anterior surgical approaches; BMI, body mass index; PSA, posterior surgical approaches.

^a^ Symptomatic arrhythmia or coronary artery disease.

The PSA group had a significantly longer symptom duration than the other two groups (*p* < 0.001). The mJOA score was 12.0 (11.0–13.0) in the ASA and PSA groups, which was significantly lower than 14.0 (14.0–14.0) in the conservative group (*p* < 0.001). Moreover, the bilateral symptom rate was the highest in the PSA group (*p* = 0.013). The involved level count was the highest in the PSA group at 3.0 (3.0–4.0) levels, and lowest in the ASA group at 1.0 (1.0–2.0) level (*p* < 0.001). Regarding the lesion type, the disc herniation rate was relatively higher in the ASA group, while the rates for OPLL, spondylolisthesis, and other/combined etiology were relatively higher in the PSA group (*p* < 0.001). The surgical treatment group showed a higher rate of HSI on T2 images, higher MC grade, and lower rate of the k-line (+) cases (*p* < 0.001, *p* < 0.001, and *p* = 0.003, respectively). Further, the surgical treatment group presented more severe CMCT deterioration and a higher rate of radiculopathy (*p* < 0.001 and *p* = 0.026, respectively) (Table 2 and Supplementary Table S3).

**Table 2:** Disease-related features.

	Conservative (*n* = 109)	ASA (*n* = 66)	PSA (*n* = 125)	*p*-value
Symptom duration, months	3.0 (2.0–6.0)	3.5 (2.0–12.0)	10.0 (3.0–24.0)	<0.001
NRS, neck	3.0 (2.0–5.0)	4.0 (3.0–6.0)	3.0 (2.0–5.0)	0.099
NRS, arm	4.0 (3.0–5.0)	5.0 (3.0–7.0)	4.0 (3.0–6.0)	0.146
mJOA score	14.0 (14.0–14.0)	12.0 (11.0–13.0)	12.0 (11.0–13.0)	<0.001
Symptom side, *n* (%)				0.013
Right	25 (22.9)	11 (16.7)	12 (9.6)	
Left	32 (29.4)	13 (19.7)	28 (22.4)	
Bilateral	52 (47.7)	42 (63.6)	85 (68.0)	
Number of involved levels	2.0 (1.0–3.0)	1.0 (1.0–2.0)	3.0 (3.0–4.0)	<0.001
Lesion type, *n* (%)				<0.001
OPLL	44 (40.4)	14 (21.2)	41 (32.8)	
Disc herniation	56 (51.4)	33 (50.0)	17 (13.6)	
Spondylolisthesis	6 (5.5)	12 (18.2)	39 (31.2)	
Others or combined	3 (2.8)	7 (10.6)	28 (22.4)	
Most stenotic level, *n* (%)				0.082
C1/2	0 (0.0)	0 (0.0)	5 (4.0)	
C2/3	2 (1.8)	0 (0.0)	1 (0.8)	
C3/4	11 (10.1)	8 (12.1)	18 (14.4)	
C4/5	19 (17.4)	20 (30.3)	30 (24.0)	
C5/6	47 (43.1)	28 (42.4)	49 (39.2)	
C6/7	30 (27.5)	10 (15.2)	22 (17.6)	
HSI on T2 image, *n* (%)	20 (18.3)	35 (53.0)	90 (72.0)	<0.001
Muhle’s classification, *n* (%)				<0.001
Grade I	33 (30.3)	4 (6.1)	4 (3.2)	
Grade II	63 (57.8)	35 (53.0)	50 (40.0)	
Grade III	13 (11.9)	27 (40.9)	71 (56.8)	
K-line (+), *n* (%)	104 (95.4)	52 (78.8)	102 (81.6)	0.003
APB-CMCT^a^, *n* (%)				<0.001
Normal	96 (88.1)	19 (28.8)	20 (16.0)	
Mildly delayed	10 (9.2)	24 (36.4)	39 (31.2)	
Definitely delayed	3 (2.8)	17 (25.8)	48 (38.4)	
Not evoked MEP	0 (0.0)	6 (9.1)	18 (14.4)	
Radiculopathy, *n* (%)	29 (26.6)	30 (45.5)	49 (39.2)	0.026

APB, abductor pollicis brevis; ASA, anterior surgical approaches; CMCT, central motor conduction time; HSI, high signal intensity; MEP, motor evoked potential; mJOA, modified Japanese Orthopaedic Association scale; NRS, numerical rating scale of pain; OPLL, ossification of the posterior longitudinal ligament; PSA, posterior surgical approaches.

^a^ Normal, CMCT <11.5 ms; mildly delayed, 11.5 ≤CMCT <15; and definitely delayed, CMCT ≥15.

### Multiclass classification

When multiclass classification was performed between the conservative, ASA, and PSA groups, the test prediction AUC-ROC of the RF and XGB models were 0.91 and 0.92, respectively. The overall accuracies of the RF and XGB models were 76.2% and 74.6%, respectively (Table 3). The confusion matrix of multiclass classification for each algorithm is presented in Supplementary Table S4.

**Table 3:** Results of multiclass classification.

Metric	RF	XGB
AUC-ROC^a^	0.91	0.92
Overall accuracy (%)	76.2	74.6

AUC-ROC, area under the receiver operating characteristic curve; RF, random forest; XGB, extreme gradient boosting.

^a^ Calculated by micro-averaging method.

### Binary classifications (one-versus-rest and one-versus-one)

The results of the binary classification of each algorithm are summarized in Table 4. The confusion matrix for each classification is presented in Supplementary Table S5.

**Table 4:** Results of binary classifications.

Classification	Algorithm	AUC-ROC	F1	AUC-PR	Sensitivity (%)	Specificity (%)	Precision (%)	NPV (%)
Conservative vs. Surgical	RF	0.94	0.93	0.96	89.6	86.7	95.6	72.2
	XGB	0.93	0.93	0.95	89.1	94.1	97.6	76.2
ASA vs. PSA	RF	0.99	0.94	0.96	100.0	88.0	76.9	100.0
	XGB	0.96	0.80	0.82	100.0	80.0	66.7	100.0

ASA, anterior surgical approaches; AUC-PR, area under the precision-recall curve; AUC-ROC, area under the receiver operating characteristic curve; NPV, negative predictive value; PSA, posterior surgical approaches; RF, random forest; XGB, extreme gradient boosting.

In the classification model between conservative and surgical treatments, RF and XGB showed AUC-ROC values of 0.94 and 0.93, respectively. The mJOA score and normal CMCT category were the two most important variables in both RF and XGB models for classifying conservative and surgical groups. Age and symptom duration were also included in the top five important features in both models (Table 5).

**Table 5:** The top five important variables in each model.

Order	Conservative vs. Surgical		ASA vs. PSA	
	Random forest	Extreme gradient boosting	Random forest	Extreme gradient boosting
1	mJOA score	mJOA score	Level count	Level count
2	Normal CMCT	Normal CMCT	Age	Age
3	HSI	Age	NRS, neck	BMI
4	Symptom duration	BMI	BMI	NRS, neck
5	Age	Symptom duration	Disc herniation lesion	Symptom duration

ASA, anterior surgical approaches; BMI, body mass index; CMCT, central motor conduction time; HSI, high signal intensity; mJOA, modified Japanese Orthopaedic Association scale; NRS, numeric rating scale of pain; PSA, posterior surgical approaches; RF, random forest; XGB, extreme gradient boosting.

The ensemble ML algorithms demonstrated outstanding performance in ASA and PSA classification; both RF and XGB showed AUC-ROC of 0.99 and 0.96, respectively. The most important variable in classifying ASA and PSA was confirmed to be level count in both the RF and XGB models. In addition, age, BMI, and subjective neck pain were identified as the major common features in both models (Table 5).

## Discussion

In this study, we suggest a strategy for therapeutic decision-making by applying an ML model in patients with DCM. Our results demonstrated that the proposed ML models showed AUC-ROC values of >0.9 in all types of classifications, proving their outstanding performance. There has been an unmet need for personalized, cost-effective surgical decisions and treatment of DCM (29). As the first attempt to utilize an ML-based approach for therapeutic selection in patients with DCM, this study is significant because we present a direction for surgical decisions using a novel method. Furthermore, we presented the possibility of a comprehensive decision process for DCM therapeutic options using various clinically available determinants, which is an advantage of the ML process, rather than relying on any specific parameter. Consequently, our ML classification models can be beneficial for spine surgeons in choosing the most effective and proper surgical approach for DCM.

Our variable importance results provide empirical information on the contributing factors that affect the treatment method of DCM at the experienced-neurosurgeon level. In addition, some of the strengths of this study are the creation of various types of classification models and analysis of the importance of these factors from multiple angles. In our ML models, the mJOA score and CMCT were the most critical factors contributing to the classification of conservative and surgical treatments. Previous studies have also attempted to identify the factors that determine conservative treatment in patients with DCM. Rhee et al. (11) suggested that functional indicators such as the mJOA score, time for a 10-meter walk, and activities of daily living could determine non-operative treatment in patients with DCM through their systematic review, which was consistent with our results. Yoshimatsu et al.'s (30) multivariable logistic regression model showed that the patient's disease duration, one of the top-five important variables for both our models, should primarily be considered when deciding conservative treatment for DCM. Meanwhile, it was reported that CMCT correlated with the degree of spinal cord compression reported in imaging findings (31, 32). CMCT has been known as one of the essential diagnostic tools in myelopathy because it reflects mechanical compression as well as the functional integrity of the motor pathway (33). In particular, ML models that distinguish the surgical treatment group from the conservative group indicated normal CMCT as the critical feature in our results.

Through ML models, this study also revealed the factors in determining the ASA and PSA in the patient group for which surgical treatment was decided. In 2019, the World Federation of Neurosurgical Societies (WFNS) spine committee recommended the surgical approaches for DCM; they reported essential features to determine between ASA and PSA, such as the number of compression levels, cervical posture, and etiology of myelopathy (34, 35). In our results, it is notable that the functional level or CMCT findings were not important features in determining the approach method in patients who had already decided on surgery. Instead, the number of levels involved was the most important contributing factor in both ML models. Age was the second important variable in the models. In addition, BMI and subjective neck pain were variables with relatively high importance in the classification model between ASA and PSA. We inferred that these could be due to the characteristic advantages and disadvantages of ASA and PSA. One of the advantages of ASA is the reduced postoperative neck pain through muscle-sparing (36). In addition, this method allows for the correction of the kyphotic curvature of the cervical spine and the removal of anterior pathology such as disc herniation (37). However, ASA is preferred only for lesions confined to less than three levels. Moreover, this approach could be more difficult in extremely obese patients (38), and severe conditions such as vascular or aerodigestive complications may also occur in rare cases (39). In contrast, one of the most significant advantages of PSA is that wide decompression is possible. Furthermore, it is particularly preferred for the treatment of cervical cord compressive lesions across multiple levels—more than three levels (40). This method can preserve the lordosis of the cervical spine; however, it is vulnerable to instability (41). However, despite considering these factors, in our ML model classifying ASA and PSA, the cervical spine alignment or presence of radiculopathy unexpectedly had lower importance. Since this study targeted a group of patients with various degenerative cervical pathologies, we inferred that the number of lesion levels played a crucial role in the decision rather than the presence of cervical kyphosis or radiculopathy. Meanwhile, since we have applied strict criteria for defining k-line (−) or concomitant radiculopathy, their proportion was measured lower than that in other studies (42, 43); therefore, we inferred the possibility that they did not show higher importance. Consequently, it is necessary to consider various factors to determine the treatment direction for patients with DCM (35). The ML models proposed in this study are significant because they can support surgeons in making the complicated decision-making process more readily, efficiently, and tailored.

This study applied ML algorithms that utilize the tree-based ensemble learning method (44). RF is an advanced tree-based ensemble model that combines multiple decision trees by the bagging algorithm and effectively reduces the variance error (45). XGB, one of the most advanced ensemble algorithms using boosting, also shows superior performance compared to other traditional ML algorithms (46). It is a gradient boosting method-based ensemble algorithm with improved scalability and performance (47). These ensemble algorithms are currently the models of choice in many ML-based clinical studies on tubular data analysis (48, 49). Thus, we utilized these two algorithms in our ML processing. In our classification models, RF slightly outperformed XGB.

This study has a few limitations. First, it was a single-center study with relatively small sample size. Therefore, the results have limited generalizability. Second, as a retrospective study, since we performed ML modeling using EHR accumulated over a relatively long duration, some variables might show ambiguity in definitions. Third, we presented a model to classify the first therapeutic decision from DCM diagnosis; however, long-term follow-up data after surgery were not provided.

We applied ML to the therapeutic decision of DCM for the first time. Despite several limitations, this study can be a basis for the generalizable clinical decision model using ML algorithms in the future and referred to as a cornerstone study for achieving this purpose for many researchers. To overcome the limitations and obtain a valid and more generalizable ML model, a large sample dataset from a systematic, multicenter, and multi-ethnic-based study is needed to utilize the advantages of ML. Moreover, the dataset should include long-term outcomes to present the prognostic results of ML-based classification models.

## Conclusion

This study presented valid and feasible ML classification models for the selection of therapeutic options in patients with DCM. Our results can provide a rational basis for human experts' clinical decisions and encourage efficient and tailored decision-making. Nevertheless, further large-scale studies are required to develop a more generalizable ML model.

## Data Availability

The raw data supporting the conclusions of this article will be made available by the authors, without undue reservation.
