# Supplementary material for: Classification of expert-level therapeutic decisions for degenerative cervical myelopathy using ensemble machine learning algorithms
Source: Front Surg. 2022 Sep 6;9:1010420. doi: 10.3389/fsurg.2022.1010420 (PMC9485547; doi:10.3389/fsurg.2022.1010420)
Supplement: Supplementary file 1 [file Presentation_1.pdf]

Classification of Expert-level Therapeutic Decisions for Degenerative Cervical Myelopathy using Ensemble Machine Learning Algorithms

Supplementary Materials

1 Supplementary Tables

1.1 **Supplementary Table 1.** Variables and definitions.

1.2 **Supplementary Table 2.** Hyperparameter tuning for each classification's best model.

1.3 **Supplementary Table 3.** Numeric variables and their *p*-values of *post-hoc* analyses.

1.4 **Supplementary Table 4.** Confusion matrix of multiclass classifications.

1.5 **Supplementary Table 5.** Confusion matrix of binary classifications.

2 Supplementary Data. R MarkDown for this study.

Supplementary table 1. Variables and definitions.

Variable	Type	Description
Age	Numerical	in years
Sex	Categorical	male or female
Body mass index	Numerical	in kg/m^2; measured at the time of admission
Insurance type	Categorical	medical-aid or national health insurance
Residential area	Categorical	urban or rural; according to the administrative distinct
Hypertension	Categorical	with or without; via patient or guardian interviews, medical records, and medication history
Diabetes	Categorical	with or without; via patient or guardian interviews, medical records, and medication history
Dyslipidemia	Categorical	with or without; via patient or guardian interviews, medical records, and medication history
Heart problem	Categorical	with or without; via patient or guardian interviews, medical records, and medication history
Degenerative lumbar disease	Categorical	with or without; via patient or guardian interviews, medical records, and medication history
Symptom duration	Numerical	in months; time from first subjective symptoms related to DCM to the index diagnosis; self-reporting
NRS, neck and arm	Numerical	subjective pain at the first visit of hospital due to DCM; self-reporting

mJOA	Numerical	enquired and evaluated by experienced neurosurgeon or physiatrist at the first visit to the hospital due to DCM
Symptom side	Categorical	right, left, or bilateral according to the symptom presentation; self-reporting
Number of involved levels	Numerical	evaluated via cervical MRI by experienced neurosurgeon and radiologist
Lesion type	Categorical	OPLL, disc herniation, spondylolisthesis, or others (including combined pathologies); evaluated by experienced neurosurgeon and radiologist
Most stenotic level	Categorical	C1/2, C2/3, C3/4, C4/5, C5/6, or C6/7; evaluated by experienced neurosurgeon and radiologist
HSI on T2 image	Categorical	with or without; evaluated by experienced neurosurgeon and radiologist
Muhle's classification	Categorical	grade I, II, or III; evaluated by experienced neurosurgeon and radiologist
K-line	Categorical	(+) or (-); evaluated via sagittal view of radiograph by experienced neurosurgeon and radiologist
APB-CMCT classification	Categorical	normal, CMCT <11.5 ms; mildly delayed, 11.5≤ CMCT <15; and definitely delayed, CMCT ≥15; evaluated by an experienced physiatrist
Radiculopathy	Categorical	with or without; evaluated via electromyography by experienced physiatrists

APB, abductor pollicis brevis; CMCT, central motor conduction time; DCM, degenerative cervical myelopathy; HSI, high signal intensity; mJOA, modified Japanese Orthopaedic Association scale; MRI, magnetic resonance imaging; NRS, numerical rating scale of pain; OPLL, ossification of the posterior longitudinal ligament.

Supplementary table 2. Hyperparameter tuning for each classification's best model.

Classification	Random forest	Extreme gradient boosting
Multiclass	ntree = 500, mtry = 35	nrounds = 1000, max_depth = 8, eta = 0.01, gamma = 0, colsample_bytree = 1, min_child_weight = 1, subsample = 1
Conservative *vs*. Surgical	ntree = 500, mtry = 6	nrounds = 2000, max_depth = 8, eta = 0.3, gamma = 0, colsample_bytree = 1, min_child_weight = 1, subsample = 1
ASA *vs*. PSA	ntree = 500, mtry = 22	nrounds = 1000, max_depth = 7, eta = 0.01, gamma = 0, colsample_bytree = 1, min_child_weight = 1, subsample = 1

ASA, anterior surgical approaches; PSA, posterior surgical approaches.

Supplementary table 3. Numeric variables and their *p*-values of *post-hoc* analyses.

Variable	Conservative *vs.* ASA	Conservative *vs.* PSA	ASA *vs.* PSA	Method
Age	0.788	<0.001	0.002	one-way ANOVA with Bonferroni's multiple comparison test
BMI	0.140	>0.999	0.033	Kruskal-Wallis test with Dunn's multiple comparisons test
Symptom duration	0.130	<0.001	0.011	Kruskal-Wallis test with Dunn's multiple comparisons test
NRS, neck	0.252	>0.999	0.111	Kruskal-Wallis test with Dunn's multiple comparisons test
NRS, arm	0.202	0.440	>0.999	Kruskal-Wallis test with Dunn's multiple comparisons test
mJOA	<0.001	<0.001	0.713	Kruskal-Wallis test with Dunn's multiple comparisons test
Number of involved levels	<0.001	<0.001	<0.001	Kruskal-Wallis test with Dunn's multiple comparisons test

ANOVA, analysis of variance; ASA, anterior surgical approaches; BMI, body mass index; mJOA, modified Japanese Orthopaedic Association scale; NRS, numerical rating scale of pain; PSA, posterior surgical approaches.

Supplementary table 4. Confusion matrix of multiclass classifications.

		Reference		
Algorithm	Prediction	Conservative	ASA	PSA
RF	Conservative	11	1	4
	ASA	4	9	2
	PSA	0	4	28
XGB	Conservative	13	2	3
	ASA	2	6	3
	PSA	0	6	28

ASA, anterior surgical approaches; PSA, posterior surgical approaches; RF, random forest; XGB, extreme gradient boosting.

Supplementary table 5. Confusion matrix of binary classifications.

Classification			Reference	
	Algorithm	Prediction	Conservative	Surgical
Conservative *vs.* Surgical	RF	Conservative	13	5
		Surgical	2	43
	XGB	Conservative	16	5
		Surgical	1	41
	Algorithm	Prediction	ASA	PSA
ASA *vs.* PSA	RF	ASA	10	3
		PSA	0	22
	XGB	ASA	10	5
		PSA	0	20

ASA, anterior surgical approaches; PSA, posterior surgical approaches; RF, random forest; XGB, extreme gradient boosting.

R MarkDown for this study

```
library(caret)
library(data.table)
library(dplyr)
library(tidyverse)
library(randomForest)
library(xgboost)
library(smotefamily)
library(MLeval)
library(moonBook)
library(doParallel)
library(dummies)
library(multiROC)

op<- read_csv("nonvsop.csv")

op$class<-as.factor(op$class)
op$sex<-as.factor(op$sex)
op$ins<-as.factor(op$ins)
op$resid<-as.factor(op$resid)
op$htn<-as.factor(op$htn)
op$dia<-as.factor(op$dia)
op$dyslip<-as.factor(op$dyslip)
op$hp<-as.factor(op$hp)
op$lumbar<-as.factor(op$lumbar)
op$type<-as.factor(op$type)
op$side<-as.factor(op$side)
op$ms_level<-as.factor(op$ms_level)
op$hsi<-as.factor(op$hsi)
op$mc<-as.factor(op$mc)
op$cmct<-as.factor(op$cmct)
op$kline<-as.factor(op$kline)
op$rad<-as.factor(op$rad)

predata<-op
nearZeroVar(predata)



findCorrelation(cor(predata[,2:8]), cutoff = .70)

st_model<-preProcess(predata[,2:8], method=c("center","scale"))

data<-predict(st_model, predata)
data=as.data.frame(data)
ohe_feats=c('sex','ins','resid','htn','dia','dyslip','hp','lumbar','type','side
','ms_level','hsi','mc','cmct','kline','rad')
```

```
dummies=dummyVars(~sex+ins+resid+htn+dia+dyslip+hp+lumbar+type+side+ms_level+hsi
+mc+cmct+kline+rad, data = data)
df_ohe <- as.data.frame(predict(dummies, newdata = data))
df_combined <- cbind(data[,-c(which(colnames(data) %in% ohe_feats))],df_ohe)
dat = as.data.table(df_combined)

table(dat$class)



dat[!complete.cases(dat),]



seed<-11
set.seed(seed)
ind<-sample(2,nrow(dat),replace = T,prob = c(0.75,0.25))
traindata<-dat[ind==1,]
testdata<-dat[ind==2,]

set.seed(seed)
traindata.smote <- SMOTE(traindata[,-1], traindata$class ,K = 5, dup_size=0)
traindata.smote <- traindata.smote$data
traindata.smote$class <- as.factor(traindata.smote$class)
table(traindata.smote$class)



traindata.smote$Newclass<-relevel(traindata.smote$class, ref = "N")
levels(traindata.smote$Newclass)



table(traindata.smote$Newclass)



traindata.smote$class=NULL

detectCores()



cl <- makePSOCKcluster(12)
registerDoParallel(cl)

ctrl <- trainControl(method="repeatedcv", number=5, repeats = 50, savePrediction
s='final', classProbs=TRUE, summaryFunction = twoClassSummary, allowParallel=TRU
E)

metric <- "ROC"

tune_grid <- expand.grid(nrounds = c(1000, 1500, 2000), eta = c(0.01, 0.1, 0.3),
```

```
 max_depth = c(6, 7, 8), gamma = 0, colsample_bytree = 1, min_child_weight = 1,
subsample = 1)

set.seed(seed)
fit.rf <- train(class~., data = traindata, method = "rf", metric = metric, trCon
trol = ctrl, tuneLength=10)
fit.rf



test_pred_rf <- predict(fit.rf, newdata = testdata)
confusionMatrix(test_pred_rf, testdata$class)



test_prob_rf<- predict(fit.rf, newdata = testdata, type="prob")
rf = data.frame(test_prob_rf, testdata$class)
yrf<-evalm(rf)



yrf$stdres



imp_rf<-varImp(fit.rf, scale = FALSE)
imp_rf



set.seed(seed)
fit.rf.sm <- train(Newclass~., data = traindata.smote, method = "rf", metric = m
etric, trControl = ctrl, tuneLength=10)
fit.rf.sm



test_pred_rf.sm <- predict(fit.rf.sm, newdata = testdata)
confusionMatrix(test_pred_rf.sm, testdata$class)



test_prob_rf.sm<- predict(fit.rf.sm, newdata = testdata, type="prob")
rf.sm = data.frame(test_prob_rf.sm, testdata$class)
yrf.sm<-evalm(rf.sm)



yrf.sm$stdres



imp_rf.sm<-varImp(fit.rf.sm, scale = FALSE)
imp_rf.sm
```

```
set.seed(seed)
fit.xgb <- train(class~., data = traindata, method = "xgbTree", metric = metric,
 trControl = ctrl, tuneGrid = tune_grid)
fit.xgb


test_pred_xgb <- predict(fit.xgb, newdata = testdata)
confusionMatrix(test_pred_xgb, testdata$class)


test_prob_xgb<- predict(fit.xgb, newdata = testdata, type="prob")
xgb = data.frame(test_prob_xgb, testdata$class)
yxgb<-evalm(xgb)


yxgb$stdres

imp_xgb<-varImp(fit.xgb, scale = FALSE)
imp_xgb


set.seed(seed)
fit.xgb.sm <- train(Newclass~., data = traindata.smote, method = "xgbTree", metr
ic = metric, trControl = ctrl, tuneGrid = tune_grid)
fit.xgb.sm


test_pred_xgb.sm <- predict(fit.xgb.sm, newdata = testdata)
confusionMatrix(test_pred_xgb.sm, testdata$class)


test_prob_xgb.sm<- predict(fit.xgb.sm, newdata = testdata, type="prob")
xgb.sm = data.frame(test_prob_xgb.sm, testdata$class)
yxgb.sm<-evalm(xgb.sm)


yxgb.sm$stdres


imp_xgb.sm<-varImp(fit.xgb.sm, scale = FALSE)
imp_xgb.sm
```

```
ap<- read_csv("antvspost.csv")

ap$class<-as.factor(ap$class)
ap$sex<-as.factor(ap$sex)
ap$ins<-as.factor(ap$ins)
ap$resid<-as.factor(ap$resid)
ap$htn<-as.factor(ap$htn)
ap$dia<-as.factor(ap$dia)
ap$dyslip<-as.factor(ap$dyslip)
ap$hp<-as.factor(ap$hp)
ap$lumbar<-as.factor(ap$lumbar)
ap$type<-as.factor(ap$type)
ap$side<-as.factor(ap$side)
ap$ms_level<-as.factor(ap$ms_level)
ap$hsi<-as.factor(ap$hsi)
ap$mc<-as.factor(ap$mc)
ap$cmct<-as.factor(ap$cmct)
ap$kline<-as.factor(ap$kline)
ap$rad<-as.factor(ap$rad)

predata<-ap
nearZeroVar(predata)



findCorrelation(cor(predata[,2:8]), cutoff = .70)



st_model<-preProcess(predata[,2:8], method=c("center","scale"))

data<-predict(st_model, predata)
data=as.data.frame(data)
ohe_feats=c('sex','ins','resid','htn','dia','dyslip','hp','lumbar','type','side
','ms_level','hsi','mc','cmct','kline','rad')
dummies=dummyVars(~sex+ins+resid+htn+dia+dyslip+hp+lumbar+type+side+ms_level+hsi
+mc+cmct+kline+rad, data = data)
df_ohe <- as.data.frame(predict(dummies, newdata = data))
df_combined <- cbind(data[,-c(which(colnames(data) %in% ohe_feats))],df_ohe)
dat = as.data.table(df_combined)

table(dat$class)



dat[!complete.cases(dat),]



set.seed(seed)
ind<-sample(2,nrow(dat),replace = T,prob = c(0.75,0.25))
traindata<-dat[ind==1,]
```

```
testdata<-dat[ind==2,]

set.seed(seed)
traindata.smote <- SMOTE(traindata[,-1], traindata$class ,K = 2, dup_size=1)
traindata.smote <- traindata.smote$data
traindata.smote$class <- as.factor(traindata.smote$class)
table(traindata.smote$class)


traindata.smote$Newclass<-relevel(traindata.smote$class, ref = "P")
levels(traindata.smote$Newclass)


table(traindata.smote$Newclass)


traindata.smote$class=NULL

set.seed(seed)
fit.rf <- train(class~., data = traindata, method = "rf", metric = metric, trCon
trol = ctrl, tuneLength=10)
fit.rf


test_pred_rf <- predict(fit.rf, newdata = testdata)
confusionMatrix(test_pred_rf, testdata$class)


test_prob_rf<- predict(fit.rf, newdata = testdata, type="prob")
rf = data.frame(test_prob_rf, testdata$class)
yrf<-evalm(rf)


yrf$stdres


imp_rf<-varImp(fit.rf, scale = FALSE)
imp_rf


set.seed(seed)
fit.rf.sm <- train(Newclass~., data = traindata.smote, method = "rf", metric = m
etric, trControl = ctrl, tuneLength=10)
fit.rf.sm
```

```
test_pred_rf.sm <- predict(fit.rf.sm, newdata = testdata)
confusionMatrix(test_pred_rf.sm, testdata$class)


test_prob_rf.sm<- predict(fit.rf.sm, newdata = testdata, type="prob")
rf.sm = data.frame(test_prob_rf.sm, testdata$class)
yrf.sm<-evalm(rf.sm)


yrf.sm$stdres


imp_rf.sm<-varImp(fit.rf.sm, scale = FALSE)
imp_rf.sm


set.seed(seed)
fit.xgb <- train(class~., data = traindata, method = "xgbTree", metric = metric,
 trControl = ctrl, tuneGrid = tune_grid)
fit.xgb


test_pred_xgb <- predict(fit.xgb, newdata = testdata)
confusionMatrix(test_pred_xgb, testdata$class)


test_prob_xgb<- predict(fit.xgb, newdata = testdata, type="prob")
xgb = data.frame(test_prob_xgb, testdata$class)
yxgb<-evalm(xgb)


yxgb$stdres


imp_xgb<-varImp(fit.xgb, scale = FALSE)
imp_xgb


set.seed(seed)
fit.xgb.sm <- train(Newclass~., data = traindata.smote, method = "xgbTree", metr
ic = metric, trControl = ctrl, tuneGrid = tune_grid)
fit.xgb.sm
```

```
test_pred_xgb.sm <- predict(fit.xgb.sm, newdata = testdata)
confusionMatrix(test_pred_xgb.sm, testdata$class)


test_prob_xgb.sm<- predict(fit.xgb.sm, newdata = testdata, type="prob")
xgb.sm = data.frame(test_prob_xgb.sm, testdata$class)
yxgb.sm<-evalm(xgb.sm)


yxgb.sm$stdres


imp_xgb.sm<-varImp(fit.xgb.sm, scale = FALSE)
imp_xgb.sm


mcl<- read_csv("multiclass.csv")

mcl$class<-as.factor(mcl$class)
mcl$sex<-as.factor(mcl$sex)
mcl$ins<-as.factor(mcl$ins)
mcl$resid<-as.factor(mcl$resid)
mcl$htn<-as.factor(mcl$htn)
mcl$dia<-as.factor(mcl$dia)
mcl$dyslip<-as.factor(mcl$dyslip)
mcl$hp<-as.factor(mcl$hp)
mcl$lumbar<-as.factor(mcl$lumbar)
mcl$type<-as.factor(mcl$type)
mcl$side<-as.factor(mcl$side)
mcl$ms_level<-as.factor(mcl$ms_level)
mcl$hsi<-as.factor(mcl$hsi)
mcl$mc<-as.factor(mcl$mc)
mcl$cmct<-as.factor(mcl$cmct)
mcl$kline<-as.factor(mcl$kline)
mcl$rad<-as.factor(mcl$rad)

mytable(class~., data=mcl, method=3)


predata<-mcl
nearZeroVar(predata)


findCorrelation(cor(predata[,2:8]), cutoff = .70)
```

```
st_model<-preProcess(predata[,2:8], method=c("center","scale"))

data<-predict(st_model, predata)
data=as.data.frame(data)
ohe_feats=c('sex','ins','resid','htn','dia','dyslip','hp','lumbar','type','side
','ms_level','hsi','mc','cmct','kline','rad')
dummies=dummyVars(~sex+ins+resid+htn+dia+dyslip+hp+lumbar+type+side+ms_level+hsi
+mc+cmct+kline+rad, data = data)
df_ohe <- as.data.frame(predict(dummies, newdata = data))
df_combined <- cbind(data[,-c(which(colnames(data) %in% ohe_feats))],df_ohe)
dat = as.data.table(df_combined)

table(dat$class)



dat[!complete.cases(dat),]



set.seed(seed)
ind<-sample(2,nrow(dat),replace = T,prob = c(0.75,0.25))
traindata<-dat[ind==1,]
testdata<-dat[ind==2,]

ctrl <- trainControl(method="repeatedcv", number=5, repeats = 50, allowParallel=
TRUE)
metric <- "Accuracy"

set.seed(seed)
fit.rf <- train(class~., data = traindata, method = "rf", metric = metric, trCon
trol = ctrl, tuneLength=10)
fit.rf



test_pred_rf <- predict(fit.rf, newdata = testdata)
confusionMatrix(test_pred_rf, testdata$class)



rf_pred <- predict(fit.rf, newdata = testdata, type = 'prob')
rf_pred <- data.frame(rf_pred)
colnames(rf_pred) <- paste(colnames(rf_pred), "_pred_RF")

true_label <- dummies::dummy(testdata$class, sep = ".")
true_label <- data.frame(true_label)
colnames(true_label) <- gsub(".*?\\.", "", colnames(true_label))
colnames(true_label) <- paste(colnames(true_label), "_true")
final_df <- cbind(true_label, rf_pred)
roc_res <- multi_roc(final_df, force_diag=F)
plot_roc_df <- plot_roc_data(roc_res)
```

```
require(ggplot2)
ggplot(plot_roc_df, aes(x = 1-Specificity, y=Sensitivity)) +
  geom_path(aes(color = Group, linetype=Method), size=1.5) +
  geom_segment(aes(x = 0, y = 0, xend = 1, yend = 1),
               colour='grey', linetype = 'dotdash') +
  theme_bw() +
  theme(plot.title = element_text(hjust = 0.5),
        legend.justification=c(1, 0), legend.position=c(.95, .05),
        legend.title=element_blank(),
        legend.background = element_rect(fill=NULL, size=0.5,
                                         linetype="solid", colour ="black"))



unlist(roc_res$AUC)



set.seed(seed)
fit.xgb <- train(class~., data = traindata, method ="xgbTree", metric = metric,
trControl = ctrl, tuneGrid = tune_grid)
fit.xgb



test_pred_xgb <- predict(fit.xgb, newdata = testdata)
confusionMatrix(test_pred_xgb, testdata$class)



xgb_pred <- predict(fit.xgb, newdata = testdata, type = 'prob')
xgb_pred <- data.frame(xgb_pred)
colnames(xgb_pred) <- paste(colnames(xgb_pred), "_pred_XGB")

true_label <- dummies::dummy(testdata$class, sep = ".")
true_label <- data.frame(true_label)
colnames(true_label) <- gsub(".*?\\.", "", colnames(true_label))
colnames(true_label) <- paste(colnames(true_label), "_true")
final_df <- cbind(true_label, xgb_pred)
roc_res <- multi_roc(final_df, force_diag=F)
plot_roc_df <- plot_roc_data(roc_res)

require(ggplot2)
ggplot(plot_roc_df, aes(x = 1-Specificity, y=Sensitivity)) +
  geom_path(aes(color = Group, linetype=Method), size=1.5) +
  geom_segment(aes(x = 0, y = 0, xend = 1, yend = 1),
               colour='grey', linetype = 'dotdash') +
  theme_bw() +
  theme(plot.title = element_text(hjust = 0.5),
        legend.justification=c(1, 0), legend.position=c(.95, .05),
        legend.title=element_blank(),
```

```
        legend.background = element_rect(fill=NULL, size=0.5,
                                         linetype="solid", colour ="black"))


unlist(roc_res$AUC)
```
